# Medicine

**Published:** 1900-09

**Authors:** G. Parker


### Progress of the Medical Sciences.

### MEDICINE.

**Typhoid fever** has produced such terrible results in our African army that its prevention and treatment are of special interest at the present time. Whatever the report of the Commission may be upon the management of the sick at Bloemfontein, no one doubts the prevalence of this scourge either in South Africa before the war, or among the troops in India, where the losses produced by it have been increasing annually for some time. In 1897 one-third of all the deaths in the British army in India were due to it. That is to say, 638 men, or about 9 per 1000 of the total strength, were killed outright, and another 1,500 were invalided for perhaps six months. Yet this is at a time when the mortality of the Indian troops from every other disease is being reduced, till their health compares very favourably with that of soldiers at home. Extraordinary efforts have been made by the government to get rid of this scourge, but in vain.

There are all kinds of theories as to the cause of the infection. It is clear that the officers and the younger men are the most susceptible, but how the infection spreads is unknown. In many instances the water supply is perfect, but still the spread of the disease goes on. The mortality, too, is terribly high—25 per cent., instead of the 4 per cent. of the German army, the 7 per cent. of American and other hospitals under the Brand treatment, and the 15 to 18 per cent. of most English institutions. In our late frontier campaigns, and among the American troops which had returned from Cuba, there were especially bad epidemics.

Professor A. E. Wright has been allowed a trial of his serum, as is well known, and the results in Africa are anxiously awaited. It is to be regretted, however, that large bodies of men were permitted to imperil the credit of the method by undergoing a single inoculation only, instead of two or three. The serum is a pure culture of the bacillus, which has been subjected to a temperature of 60 centigrade. Animals thus inoculated become highly resistant to infection, and in human beings the same Widal reactions appear as are seen after an attack of ordinary typhoid, and last for at least a year or two, and possibly much longer. After a few trials in the Soudan, 2,855 men were each inoculated once in India,[1] with the result that only .95 per cent. were attacked, instead of 2.5 in the uninoculated, or a reduction of two-thirds of the usual number of cases; while the mortality of those attacked among the inoculated was little more than half that among the others. These figures, too, do not represent the full gain, since the inoculated were new-comers, in whom the rates would usually be much higher. At present nothing can be learnt as to the success of the method from the fragmentary reports which have come from Africa.

The report of a commission to the American Government[2] shows that everyone of their regiments in 1898 were attacked by typhoid soon after they went into camps, and the facts showed once more that the contagion did not arise *de novo*, but depended on a specific germ which was conveyed from one patient to another. The chief modes of infection seemed to be by flies, dust, and personal contact, rather than by contaminated water supplies. Indeed, a regiment appeared to carry the disease with it across the ocean when their clothing and belongings had become infected.

The urine of typhoid patients is a frequently over-looked source of infection. W. H. Park showed sometime ago that the bacilli exist in it for weeks after convalescence, and recommended urotropine as a means of destroying them. They are not found for, perhaps, the first twenty days of the illness, nor do they occur in the urine of every patient; but they are a

[1] *Brit. M. J.*, 1900, i. 122. [2] *Phila. M. J.*, 1900, v. 1315.

serious danger to others in the many cases where they do exist, and they frequently produce a form of cystitis in the patient himself. Horton-Smith[1] points out that urotropine gets rid of the bacilli and the cystitis, making the urine clear in twenty-four hours. It has little effect in the acid cystitis due to the bacillus coli, but in typhoid or ammoniacal cases the results are rapid and certain. A dose of ten grains given three times a day for a week, and sometimes for a shorter period, gets rid of the bacilli completely. No such remedy has been found for the infection of the gall-bladder, which is said to occur almost universally,[2] and may last for twelve or fifteen years with or without symptoms. Various forms of local trouble may result, such as cholecystitis, perforation, gangrene; and possibly even gall-stones, it is thought, are often deposited upon a clump of bacilli as a nucleus.

Two startling and remarkable changes in the **treatment of typhoid** have been advocated by several thoughtful writers, though their arguments are not convincing. The first is the use of a fuller diet which Barr, Shattuck, and others have advised. Bushuyev treated 318 patients with apparently good results on a much relaxed diet, and Marsden, at the Monsall Fever Hospital, allowed the milder cases in a group of 200 to go on to fish, bread, chicken, and minced meat as soon as the appetite and tongue improved. Half his cases were on fish before the temperature was normal. No perforation and few hemorrhages occurred, while recoveries were rapid and with few complications. Manges[3] gives an able review of the arguments in favour of a change. The other point is the permission for mild cases to step in and out of the bath themselves. This method has been adopted by some American and Australian authorities. Wilson and Salinger[4] at the German Hospital in Philadelphia give a bath whenever the temperature reaches 101.4°, and the less severe cases walk from their beds as often as necessary. In no instance have bad effects been observed by them. We may add that their paper shows incidentally the enormous value of the Brand system itself, the proof of which lies on a very different basis. They discuss 1904 cases treated in Philadelphia hospitals with a mortality of only 7.5 per cent. Hare, of Brisbane, records the same mortality in about the same number of cases. Thompson, of the Roosevelt Hospital, shows only 6.8 per cent. in 368 patients, using, at least, of late, artificial Nauheim water for the bath; and the Bellevue Hospital 9.6 per cent.

The boundaries of the **rheumatic group of affections** are continually changing. Many diseases formerly included are

[1] Goulstonian Lecture, 1900; *Brit. M. J.*, 1900, i. 827.

[2] *Brit. M. J.*, 1900, i. Epitome, p. 93.

[3] *Med. Rec.*, 1900, lvii. 1. [4] *Phila. M. J.*, 1900, v. 510.

now turned out and others are taken in. Thus the septic, gonorrhœal, tabetic, and rheumatoid forms of arthritis are seen to be distinct, while certain febrile states in children and adults with endocarditis are clearly rheumatism even if no joint troubles exist. A third class, including many of the skin affections, chorea and chronic joint affections, takes an uncertain place according to the views of individual authors. Thus muscular rheumatism, a variety with very scanty pathology, has been placed in a fresh light by Leube, and Adler.[1] The latter lays down that the essential lesion here is an interstitial myositis due to the toxin of true rheumatism. This applies both to acute and mild forms, and both are, he finds, followed at times by endocarditis, and skin lesions such as peliosis and urticaria. Cases which come on after an injury are really the same, for here a patch of myositis has existed without symptoms until irritated by the strain. In persons who are subject to recurrent attacks, the infiltrated patches or nodules can be easily felt, and when pain commences it is situated in these spots. Indurated patches may also be found sometimes in persons who have had cardiac troubles without joint affections. As to treatment the writer recommends salol, and the use of an ice-bag, together with careful massage after the acute stage is over. A skilful masseur can often detect these masses of induration in unsuspected quarters such as the rectus abdominis, or even the scalp, when the large superficial muscles of the back are free from them.

In speaking of treatment, we may allude to a method which is extremely useful in some cases of lumbago and of sciatica. Pure strong hydrochloric acid is painted on the skin in parallel lines by a glass or wooden rod. A surface of perhaps three inches square over the course of the nerve, or where the pain is most severe, is thus treated, allowed to dry, and then covered with cotton wool. No irritation or soreness of the skin is usually felt, and the painting can be repeated for several days. A large number of cases are found to be cured after a few applications, and others are relieved.

A. P. Luff[2] acknowledges that there is an affection of the muscles in true rheumatism, and he employs it as one of the diagnostic signs for separating that disease from rheumatoid arthritis and gout. The other positive signs of rheumatism on which he relies are: the way in which the attack flies from joint to joint, while those first attacked rapidly become normal again, without bony outgrowths or lipping of cartilages; the frequent presence of skin affections; and the improvement following the use of salicylates.

On the other hand, the means of distinguishing rheumatoid arthritis are daily becoming more complete. The labours of Wohlmann and Bannatyne on its pathology seem to have been

[1] *Med. Rec.*, 1900, lvii. 529. [2] *Practitioner*, 1900, lxiv. 491.

at last completed by the reproduction of the disease in animals. Von Dungern and Schneider[1] obtained a small diplococcus from the joints and gall-bladder of a patient. This they cultivated and injected into the joints of animals, with the result that the lesions found in the original patient were reproduced. Other organisms in control experiments produced suppuration, but erosion of cartilages was the work of these diplococci alone. It is, however, probable that there is more than one variety of the disease, and even a non-bacillary form, the mono-arthritis of the aged, which presents similar symptoms to true arthritis. An attempt at classifying the various types of this disorder has been made by W. Armstrong on a causal basis. Thus he groups together the cases which occur after gout, or true rheumatism: those which appear in phthisis, after uterine disease, after much intestinal fermentation, and those in nerve diseases. There is much difference between some of these types in reaction to treatment, but the difference is not unlikely to depend on the resisting power of the sufferers rather than on specific varieties in the disease. Clinically, we may make a useful classification from the schemes put forward by Garrod,[2] Bannatyne and others :—(1) A form seen in children with enlargement of the spleen and glands. There is a symmetrical affection of many joints which show neither anchylosis nor bony outgrowths, though there is great thickening of the tissues around the joints.[3] (2) A more or less acute type, most common in young women, and often following some acute infectious disorder. This is called the "fusiform" variety, from the shape of the joints of the hands, which are attacked symmetrically and show much effusion but no bony outgrowths. The terminal joints of the fingers escape, while the neck and, for a time at least, the temporo-maxillary joints are involved. There is much muscular wasting, but no bony anchylosis takes place. (3) "The crippling form" commences often in early adult life, and here many or all of the joints in the body tend to become rapidly attacked one after the other, and to be completely fixed. Large bony outgrowths and remarkable deformities result, and the prognosis is of the gloomiest kind. (4) "The nodular form," seen in elderly persons, especially in women, progresses but slowly, and often begins in the ball of the thumb. Especially noticeable are the bony enlargements of the terminal phalanges of the fingers, or Heberden's nodes, which are never seen in the fusiform type. The other phalanges and larger joints are often involved, but here, too, we find chiefly changes in the bones rather than in the soft tissues, yet anchylosis is rare. These are the cases which used to be called chronic rheumatism of old people. (5) Senile osteo-arthritis, affecting only one or two joints, preferably the hip, and often

[1] *München. med. Wchnschr.*, 1898, xlv. 1369. [2] *Practitioner*, 1900, lxiv. 514

[3] G. F. Still, Allbutt's *System of Medicine*, vol. iii., 1897, p. 102.

coming on after a blow, is probably quite distinct in origin and most unsatisfactory as to prognosis.

An early recognition of the disease which we have to do with is of the greatest importance, since both the food and medicine desirable differ from those in true rheumatism or in gout. In arthritis it is sometimes possible to discover the source of the infection, *e.g.*, a uterine erosion or a chronic intestinal fermentation. If these can be removed and the patient placed on an abundant nitrogenous diet, with cod-liver oil, iodide of iron, tonics, and hot-air baths, the disease may sometimes be arrested altogether. Garrod's view is probably correct that the most hopeful variety is the fusiform, where satisfactory results are not uncommon if the treatment is well chosen and continued for a long period.

It seemed not long ago that we were reaching some kind of settled agreement as to the origin of **gout,** thanks to the labours of Garrod, Roberts, and others; but every branch of the question has been re-opened recently. Thus uric acid was thought to be formed in the kidneys by a sort of perverted metabolism; but Horbaczewski pointed out a source in the nuclein of broken-down leucocytes, which raised the question why leukæmia does not cause gouty symptoms since the amount of uric acid formed is so great. The answer given was that gout is due to the retention of uric acid, while with healthy kidneys any excess in leukæmia or similar states is got rid of. Vaughan Harley, however, showed that the relation between an increase of white cells and of the uric acid excreted even with healthy kidneys is not fixed. Then it was found that uric acid could come from the nuclein in the food consumed, and unfortunately for the retention theory a gouty patient to whom thymus gland containing much nuclein was given showed an increased excretion of uric acid. W. J. S. Jerome[6] and others go further, and derive it not only from nuclein in the food, but also from hypoxanthin and other alloxuric bodies also found in the food. Thus it is increased by a meal of fishes' roe or Liebig's extract. C. C. Douglas[7] indeed would deny its origin from leucocytes at all, pointing to the fact that there is no increased excretion of phosphoric acid accompanying that of uric acid, which should be the case if leucocytes were the source. Halliburton[8] in his late introductory address laid stress on the common radicle purine possessed both by uric acid and the alloxuric bodies, while Ebstein at Paris questioned the importance in gout of the uric acid which was derived from the food taken. Indeed, A. C. Crofton[9] goes further still, and considers that uric acid, from whatever source it is derived, is quite harmless, except from

[6] *J. Physiol.*, 1899–1900, xxv. 98, and *Brit. M. J.*, 1900, i. 32.
[7] *Edinb. M. J.*, 1900, N.S. vii. 32. [8] *Lancet*, 1900, ii. 447.
[9] *British Physician*, March 15, 1900.

the risk of its forming concretions, that in uric acid we have the safest mode of getting rid of broken-down nuclein from the system, and that the harm usually attributed to it is caused by alloxuric bodies. Indeed, he has actually brought about kidney lesions, such as may be found in gout, by injecting some of these bodies, while uric acid introduced in the same way produced no such effect. Minkowski also by administering adenin, which is akin to hypoxanthin, produced a crystalline deposit in the kidneys. This view is however consistent with the theory that uric acid is a mechanical cause of the joint troubles in gout. Indeed, Freudweiler[1] claims to have produced local changes similar to those in gout by injecting uric acid into the tissues.

In other branches of the subject similar activity has been shown. A. P. Luff[2] has finally disposed of the old idea that the blood in gout shows acidity, or is at least less alkaline than usual. As a matter of fact, he finds the alkalinity is a third higher than normal; and as it is due of course to sodium bicarbonate, which favours the deposition of urate of soda crystals, we can understand why attacks are prone to occur in such persons. Luff goes on to argue that uric acid occurs in the blood in three forms—an unstable quadriurate, a gelatinous biurate, and then a crystalline and insoluble biurate. If we can delay the change from the gelatinous to the crystalline form while elimination is going on, the irritant matter may be got rid of altogether. Such a delay is produced by potassium salts, and to some extent by lithium and piperazine. Vegetables such as spinach, winter greens, turnips, celery, and French beans also delay this conversion, and are therefore useful in gout. The harm done by certain wines is not due to any supposed "acidity," but to some irritation of the liver which they cause. The value of colchicum, on the other hand, depends on its power of lessening the formation of uric acid—another fact which has been observed but not explained. Guaiacum resin, according to Bain's[3] experiments, greatly increases the excretion of uric acid, and is recommended in twenty grains doses twice a day for a fortnight at a time. A rather serious attack on the theory of quadriurates has been made by Tunnicliffe and Rosenheim,[4] which, if proved, would render doubtful much that has been asserted. They maintain that quadriurates are merely varying mixtures of uric acid and biurates, and when prepared according to Roberts' direction, and analysed, the composition is irregular. The important thing is that uric acid in this form is a freely soluble substance, but at a later stage it tends to precipitate in crystalline form. If, as Luff says, it is possible to put off the

[1] *Am. J. M. Sc.*, 1900, cxix. 459. [2] *Brit. M. J.*, 1900, i. 836.
[3] *Lancet*, 1900, i. 1444, and *Brit. M. J.*, 1900, i. 834.
[4] *Lancet*, 1900, i. 1708.

moment of crystallisation and to get rid of the poison in the meantime, we have saved the patient from an attack.

A method of treatment for **burns produced by carbolic acid** seems worth recording here. Phelps and Powell[1] believe that these injuries can be prevented by quickly bathing the parts in strong alcohol, and to some extent this is true. Pure carbolic acid can be poured, for instance, over the hands without harm if they are at once washed in alcohol, not because the alcohol forms any chemical compound with the carbolic, but probably because it washes it away completely. It has been recommended to wash out the stomach with alcohol when carbolic has been taken by the mouth, and some remarkable recoveries have followed its use.

G. PARKER.

